# Manifestations of personal characteristics in individual oral care

**DOI:** 10.1186/s13167-016-0058-2

**Published:** 2016-04-15

**Authors:** Vadim V. Tachalov, Lyudmila Y. Orekhova, Tatyana V. Kudryavtseva, Elena R. Isaeva, Ekaterina S. Loboda

**Affiliations:** 1 City Periodontology Center “PAKS”, Therapeutic Dentistry Department, Pavlov First Saint Petersburg State Medical University of the Ministry of Health of the Russian Federation, Marata St. 41-20, Saint Petersburg, 191002 Russia; 2 General and Clinical Psychology Department, Pavlov First Saint Petersburg State Medical University of the Ministry of Health of the Russian Federation, Saint Petersburg, Russia

**Keywords:** Prevention of dental diseases, Oral care, Dental status, Psychological status, Motivation for individual oral care

## Abstract

**Background:**

Preventing diseases of oral cavity, specifically periodontal diseases, is currently a high-priority issue. Despite the wide selection of individual oral care products and the abundance of information on individual oral care, the prevalence of tooth decay and inflammatory periodontal diseases remains high. Apart from knowledge of individual oral care, of great importance for patients is their capacity to develop strong motivation for full-scale hygienic procedures.

**Results:**

Patients’ motivation is immediately interconnected with their personality specifics.

**Conclusions:**

This article provides the analysis on the relationship between the patient’s oral cavity status and his or her personality profile.

## Overview

The “doctor-patient” relationship is one of the most important components of the medical dental care activity system [1]. Given the severe competition between dental clinics, apart from manual skills, dentists need the knowledge of psychological characteristics of their patients, because the relationships in the dyad doctor-patient are crucial for the success of the treatment and the implementation of doctor’s recommendations for the patient [2].

As a rule, the time for the dental visit is strictly limited, so the doctor does not always pay sufficient attention to psychological characteristics of patients. Therefore, doctors often do not use the opportunity of emotional regulation of their patients in account of their individuality as a whole, and this is what the success of dental treatment depends on [3].

## Background

The dentist should not expect the success of their work without applying individual psychological approach to each particular patient [4]. Individual motivation plays an important role in implementing recommendations on individual oral hygiene given by the attending physician [5, 6]. Numerous studies have been devoted to the effectiveness of the physician-patient interaction methods; however, none of them have identified the best model of this interaction [7].

## Methods

The study was housed by the Therapeutic Dentistry Department of the First Saint Petersburg State Medical University (SMU). A total of 153 people, 18 to 24 years of age, participated in the study, with the majority represented by students of SMU Faculty of Dentistry, who went through identification of dental status—through determining oral hygiene indexes (Greene and Vermillion, Silness and Loe, Fyodorov and Volodkina). The study also included determining the gingival bleeding index (Saxer and Muhleman) and the papillary marginal alveolar (PMA) index.

Afterwards, the patients were provided with professional hygiene, individual oral care training, and recommendations on the use of individual oral care products.

In a month, the patients were invited for a repeated examination, which included determination of dental indexes.

We developed a questionnaire to obtain information about the patient’s age, sex, social status, attitudes to his/her general health status, and dental health. To identify personality traits of the respondents, we performed a survey, which included the following psychological methods: the Leary test, big five (Big5) test, integrative anxiety test (IAT), and level of subjective control (LSC) test.

The technique of T. Leary [8] “diagnostics of interpersonal relations” (DIR, adapted from L. Sobchik) [9] is intended to define the style of interpersonal relationships, which a patient performs in relation to other people, in particular, in a situation of treatment. The following two factors are most often identified in the studies on interpersonal relationships: domination-submission and friendliness-aggressiveness. This technique will allow exploring the influence of behavioral characteristics of the patient and the style of relationships set with the physician on the implementation of medical recommendations and treatment. The questionnaire contains 128 value judgments.

The LSC questionnaire technique is a modified version of the questionnaire of the American psychologist J. Rotter [10, 11] that allows estimating the level of subjective control over a variety of situations, that is, determining the extent of human responsibility for their actions and their lives. It was established that the locus of control (responsibility) type the person belongs to affects various characteristics of his behavior. The level of subjective control is one of the important characteristics of self-awareness, which generates a sense of responsibility and willingness to be active. The LSC questionnaire consists of 44 items.

The ITA [12, 13] allows estimating the patient’s emotional status and, in particular, the level of anxiety, both as a condition and a personal characteristic.

The Big5 technique (BIG 5, Big Five Inventory) [14], adapted from A.G. Shmelev [15], is intended for express diagnostics of the patient’s main personality characteristics, the so-called big five factors of his/her temperament and character. The human personality includes five general and relatively independent features (reports): extraversion, friendliness, conscientiousness, neuroticism, and willingness to cooperate. The technique allows building a personality profile of the patient and, to some extent, predicting his/her behavior in the process of treatment.

Our research was approved by the Ethical Committee of the First Pavlov State Medical University of Saint-Petersburg.

We have obtained informed consent from each participant.

### Statistics

The main approach to the analysis of obtained data was correlation analysis using nonparametric Spearman’s rank correlation coefficient *r*_S_. Correlations were claimed as statistically significant at the commonly used significance level α = 0.05. As usual values, 0.0 < *r*_S_ < 0.4 were interpreted as practically (clinically) negligible, 0.4 < *r*_S_ < 0.6 as weak, 0.6 < *r*_S_ < 0.8 as moderate, and 0.8 < *r*_S_ < 1.0 as a strong correlation.

## Results and Discussion

The correlation analysis revealed correlations (in account of the significance level not exceeding *p* < 0.05) between psychological parameters and indexes determining the status of the oral cavity and dental behavior. The results are shown in Table 1.

**Table 1:** Correlations between scales of psychological techniques reflecting the personality and dental indexes

	Saxer 1	Saxer 2	OHI-S 1	OHI-S 2	Silness 2	The need for cleaning teeth	Correct teeth cleaning	The frequency of teeth cleaning	The frequency of changing toothbrushes	The frequency of visits to dentist	Toothbrush + mouthwash
Type of relationships with others (technique of T. Leary)											
Authoritarian							−0.41				
Selfish							−0.47		0.47		
Suspicious			0.44								
Dependent											0.41
Friendly					−0.44						
Altruistic									0.63		
Locus of control: internality/externality (LSC technique)											
In—area of failures										−0.41	
Is—family relations	0.47										
Ip—production relationships	−0.41	−0.46									
Im—interpersonal relationships						0.46			−0.40		
Situational (c) and personal (l) anxiety (IAT methodology)											
Emotional discomfort (c)									0.46		
Asthenic component (c)											
Phobic component (c)			−0.47								
Social protection (c)			−0.59	−0.55							
Overall score of anxiety (c)											
Asthenic component (l)											
Phobic component (l)				−0.45				−0.40			
Social protection (l)			−0.41								
Personal property (the “Big5” technique)											
Emotional stability									−0.42		

The statistically significant (at the significance level *α* = 0.05) values of Spearman’s correlation coefficient are shown only

*IAT* integrative anxiety test, *LSC* level of subjective control

Thus, the type of relationships with others (technique of T. Leary) was closely associated with clinical indicators of dental hygiene:

“Suspicious” type of relationships positively correlated (*r*_S_ = 0.44) with hygienic index of Greene-Vermilion (OHI-S 1) that was measured at initial examination of the patient. I.e., people with intensive manifestation of such character traits as suspiciousness and aloofness are worse in watching after their teeth and are not paying sufficient attention to the hygiene of oral cavity, which is reflected in the high rates of tartar and plaque.

“Friendly” type of relationships had negative correlation (*r*_S_ = −0.44) with Silness 2 index (determination of soft dental plaque in the gingival margin area), which was measured at the repeated examination of the patient. In other words, the repeated examination revealed positive dynamics in relation to this index in those patients who had expressed such character traits as orientation for social approval, cooperation, and flexibility in relations with others. They began to improve their teeth cleaning practices after talking with the doctor and attending a session of preventive teeth cleaning. As a result, the repeated visits revealed less plaque. Conversely, the dynamics related to the status of oral cavity and teeth were worse in patients with low levels of friendliness and openness.

“Authoritarian” type of relationships was in negative correlation with correctness of teeth cleaning practices used by patients (*r*_S_ = −0.41). In other words, people showing such character traits as dominance, leadership, and self-confidence, often have misconceptions about toothbrushing.

“Selfish” type of relationships with others was positively associated with the frequency toothbrush replacement (*r*_S_ = 0.47) and negatively associated with the correctness of toothbrushing (*r*_S_ = −0.47). In other words, people with such traits as egotistical, narcissistic, and independent, and those who neglect interests of others, take care of timely replacement of the toothbrush but have poor understanding of how to clean their teeth properly.

The study revealed positive correlation (*r*_S_ = 0.41) between the “dependent” type of relationships and the scale in the questionnaire for patients “use of toothbrush and mouthwash for oral hygiene.” Patients with dependent type in relationships (uncertainty, obedience, fearfulness, reliance on the views of others) pay more attention to their oral health and toothbrushing. They use a toothbrush and rinse more often than others.

“Altruistic” type of relationships with others was positively linked (*r*_S_ = 0.63) with the scale “the frequency of changing toothbrushes,” i.e., patients with high social responsibility, care, tenderness, and compassion in dealing with others show sufficient consideration for their hygiene and toothbrushes: they change brush regularly once every 2 months.

Therefore, the style (type) of interpersonal relations is manifested in care for the dental health and personal hygiene. “Altruistic,” “dependent” types paid more attention to toothbrushing and oral hygiene. “Friendly” type was more inclined to cooperate with the doctor: after professional tooth cleaning they started to improve their teeth cleaning practices and monitor oral hygiene, which led to a decrease in plaque. “Selfish” type also began to replace a toothbrush more frequently but had no idea of how to clean their teeth properly. “Suspicious” and “authoritarian” types showed the worst oral hygiene practices; they had not realized the need to clean their teeth properly.

Locus of control of the individual (the LSC technique) also turned out to be interconnected with clinical indicators of oral hygiene and dental behaviors*.*

The scale of “internality in the area of failures” (In) had negative correlation (*r*_S_ = −0.41) with frequency of dental visits. In other words, people with high internality (internal locus of control) attend a dentist less frequently.

The scale of “internality in family relations” (Is) had a positive correlation (*r*_S_ = 0.47) with the Saxer 1 index measured at the primary examination of the patient, i.e., the higher internality in family relations, which in our study was most often observed in men, the worse condition of oral cavity, and more intensive bleeding and inflammation of the gums.

The scale of “internality in production relationships” (Ip) was negatively related to index Saxer 1, measured at the first inspection (*r*_S_ = −0.41) and index Saxer 2 measured at repeated inspection (*r*_S_ = −0.46). I.e., personal internality (internal locus of control) in the area of work and industrial relations was accompanied by greater care of oral hygiene; it resulted in the decrease in gum inflammation.

The scale of “internality in the field of interpersonal relationships” (Im) had a positive correlation (*r*_S_ = 0.46) with the scale “the frequency of toothbrushing” and a negative relationship (*r*_S_ = −0.40) with the scale of the frequency shifts of the toothbrush. People with high internal responsibility in dealing with others are increasingly aware of the need for oral hygiene, but, on the other hand, people with externality (external locus) often replace the toothbrush.

Thus, high internality of personality (internal locus of control) has contributed to greater care of oral health, awareness of the need to clean teeth regularly, which resulted in the decrease of gum inflammation, but “internal” patients rarely went to the dentist. People with low internality (external locus of control) often change the brush.

The study also found correlations between such psychological characteristics of personality as anxiety (IAT methodology) and clinical indicators of dental health and oral hygiene.

The scale of “situational emotional discomfort” (alarm component) had a positive correlation (*r*_S_ = 0.46) with a frequency of the toothbrush replacement, i.e., patients with irritability and emotional dissatisfaction (in psychological status) often replace the brush.

Phobic component of situational anxiety negatively correlated (*r*_S_ = −0.47) with an index of OHI-S 1, which was measured at the primary examination of the patient. In other words, the more anxious the patients were, the better they cleaned their teeth; they had less plaque and tartar.

The scale of “social protection” (as a component of situational anxiety) also had negative correlation with the index of OHI-S 1 measured at the initial examination of the patient (*r*_S_ = −0.59) and at repeated examination (*r*_S_ = −0.55). Patients’ anxiety contributed to improvement of their tooth-cleaning practices, and they had less plaque.

Phobic component of personal anxiety had negative correlation with the index of OHI-S 1 (*r*_S_ = −0.45). Also phobic component correlated negatively with the frequency of toothbrushing (*r*_S_ = −0.40). The scale of “social protection” of personal anxiety had a negative correlation (*r*_S_ = −0.41) with an index of OHI-S 1, measured at the primary examination of the patient. This again confirms the above facts: personal anxiety contributed to the fact that patients cleaned their teeth better and better monitored the oral cavity (plaque and tartar became less). However, these patients brushed their teeth not so often.

Thus, anxiety in the structure of nature (situational and personal) has a significant impact on dental behavior of patients and health of the oral cavity. Anxious patients better complied with the doctor’s advice.

Negative correlation was found (*r*_S_ = −0.42) between the scale of “emotional stability” and the frequency of toothbrush replacement, i.e., the more expressed was emotional stability and balance in the structure of personality (i.e., low neuroticism), the less frequently people changed their toothbrush.

Unfortunately, all correlations revealed as statistically significant appeared to be clinically negligible or very weak with values *r*_S_ from 0.41 to 0.63 (Table 1).

Nonetheless, the results could be crucial for further search of favorable psychological factors that increase the patient’s motivation for treatment, as well as for the prognosis of the expected response to further treatment. This would differentiate (evidence-based) medical efforts and impact targets for different types of patients, depending on their personal capacity, level of compliance, and attitude towards their health and treatment. It can also help in predicting and preventing dental and periodontal diseases, so that we can save economic resources.

## Conclusions

1. People with “altruistic” and “dependent” type of interpersonal relations pay much attention to the status of their oral cavity; they address issues of hygiene and cleaning teeth with special care. To the same extent careful about the health of their teeth are people of “selfish” type, but they do not always know how to properly clean their teeth; such patients should contact a dentist for a consultation on proper oral care. People with internal locus of control also pay much attention to the status of their oral cavity.
2. The poorest oral care practices are revealed in patients of “suspicious” and “authoritarian” types of interpersonal relationships. People with external locus of control pay less attention to oral hygiene but more often replace their toothbrush.
3. Patients of “friendly” type are more than others inclined to cooperate with the doctor and comply with his recommendations; “anxious” patients also often follow the doctor’s prescriptions.

### Expert recommendations

The study showed that there are specific relations between psychological characteristics of patients and their attitude to their dental health and hygiene of oral cavity. These characteristics with a fairly high probability influenced the behavior of dental patient after the prophylactic examination and conversation with the doctor. Therefore, it is necessary to develop more specific programs of medical interaction and psychological support of different types of patients, in account of their individual psychological profile, to enhance the effectiveness of treatment and prevention activities. The results are crucial for further search of favorable psychological factors that increase the patient’s motivation for treatment, as well as for the prognosis of the expected response to further treatment. This would differentiate (evidence-based) medical efforts and impact targets for different types of patients, depending on their personal capacity, level of compliance, and attitude towards their health and treatment.

## Abbreviations

Big5: big five
IAT: integrative anxiety test
LSC: level of subjective control
OHI-S: index of Greene-Vermilion
PMA: papillary marginal alveolar
SMU: First Saint Petersburg State Medical University

## References

[1] Kelley JM, Kraft-Todd G, Schapira L, Kossowsky J, Riess H (2014). The influence of the patient-clinician relationship on healthcare outcomes: a systematic review and meta-analysis of randomized controlled trials. PloS One.

[2] Fortin AH, VI, Dwamena F, Frankel R, Smith RC. Smith’s Patient-Centered Interviewing: An Evidence-Based Method. Third ed. New York: McGraw-Hill; 2012.

[3] Pichugina EN, Arushanyan AR (2014). Individual approach to the treatment of dental patients according to the profile of their psychological status. Bull Med Internet Conf (ISSN 2224‐6150).

[4] Ayer WA. Psychology and dentistry: mental health aspects of patient care. New York: Haworth Press; 2005; 148.

[5] Asimakopoulou K, Newton JT (2015). The contributions of behavior change science towards dental public health practice: a new paradigm. Community Dent Oral Epidemiol.

[6] Asimakopoulou K, Newton JT, Daly B, Kutzer Y, Ide M (2015). The effects of providing periodontal disease risk information on psychological outcomes—a randomized controlled trial. J Clin Periodontol.

[7] Newton JT, Asimakopoulou K (2015). Managing oral hygiene as a risk factor for periodontal disease: a systematic review of psychological approaches to behavior change for improved plaque control in periodontal management. J Clin Periodontol.

[8] Leary T (1957). Interpersonal diagnosis of personality: a functional theory and methodology for personality evaluation.

[9] Sobchik LN (1990). Diagnostika mezhlichnostnykh otnosheniy: modifitsirovannyy variant interpersonal’noy diagnostiki T. Liri: metodicheskoye rukovodstvo. Seriya “Metody psikhologicheskoy diagnostiki”, Vypusk 3.

[10] Bazhin EF, Golynkina EA, Etkind AM (1993). Oprosnik urovnya sub”yektivnogo kontrolya.

[11] Rotter JB. Generalized expectancies of internal versus external control of reinforcements. Psychol Monogr. 1966; 80(1): 1–28. 5340840

[12] Spielberger CD, Gorssuch RL, Lushene PR, Vagg PR, Jacobs GA (1983). Manual for the State-Trait Anxiety Inventory.

[13] Spielberger CD, Sydeman SJ, Maruish ME (1994). State-Trait Anxiety Inventory and State-Trait Anger Expression Inventory. The use of psychological testing for treatment planning and outcome assessment.

[14] Shmelev AG. Psikhodiagnostika lichnostnykh chert. St. Petersburg, 2002. 480 p. ISBN 5-9268-0084-6 - Russian.

[15] John OP, Donahue EM, Kentle RL (1991). The Big Five Inventory—versions 4a and 54.

